# Reduced mitochondrial DNA copy number is a biomarker of Parkinson's disease

**DOI:** 10.1016/j.neurobiolaging.2015.10.033

**Published:** 2016-02

**Authors:** Angela Pyle, Haidyan Anugrha, Marzena Kurzawa-Akanbi, Alison Yarnall, David Burn, Gavin Hudson

**Affiliations:** a Mitochondrial Research Group, Institute of Genetic Medicine, University of Newcastle Upon Tyne, UK; b Insitutute of Neuroscience, University of Newcastle Upon Tyne, UK

**Keywords:** Parkinson's disease, Mitochondria, Biomarker

## Abstract

Like any organ, the brain is susceptible to the march of time and a reduction in mitochondrial biogenesis is a hallmark of the aging process. In the largest investigation of mitochondrial copy number in Parkinson's disease (PD) to date and by using multiple tissues, we demonstrate that reduced Parkinson DNA (mitochondrial DNA mtDNA) copy number is a biomarker for the etiology of PD. We used established methods of mtDNA quantification to assess the copy number of mtDNA in n = 363 peripheral blood samples, n = 151 substantia nigra pars compacta tissue samples and n = 120 frontal cortex tissue samples from community-based PD cases fulfilling UK-PD Society brain bank criteria for the diagnosis of PD. Accepting technical limitations, our data show that PD patients suffer a significant reduction in mtDNA copy number in both peripheral blood and the vulnerable substantia nigra pars compacta when compared to matched controls. Our study indicates that reduced mtDNA copy number is restricted to the affected brain tissue, but is also reflected in the peripheral blood, suggesting that mtDNA copy number may be a viable diagnostic predictor of PD.

## 1 Introduction

Parkinson's disease (PD) is a prototypical age-related neurodegenerative disease, affecting approximately 1% of the worldwide elderly population. The etiopathogenesis of PD is complex and multifactorial, including contributions from environmental and genetic factors, with aging remaining the strongest risk factor.

Sporadic PD was first linked to mitochondrial function in the late 1970's, when the potent respiratory chain inhibitor 1-methyl-4-phenyl-1,2,3,6-tetrahydropyridine was shown to cause parkinsonism in illicit drug users (Davis et al., 1979). Subsequent studies, focusing on complex I-mediated reactive oxygen species formation link PD to a vicious circle of oxidative stress and bioenergetic failure. Moreover, there is compelling evidence that mitochondrial quality control and stress responses are affected by PD-associated genes (Hatano et al., 2004; Mills et al., 2008; Mullin and Schapira, 2013). At the cellular genetic level, reports have indicated that mitochondrial DNA (mtDNA) deletion formation may contribute to etiology (Reeve et al., 2013); however, these isolated findings cannot fully explain the gross neuronal loss seen in PD. Conversely, comprehensive population studies, focusing on the role of inherited mtDNA variants, have identified phylogenetic clades which reproducibly affect PD risk (Ghezzi et al., 2005, Hudson et al., 2013, Hudson et al., 2014, Latsoudis et al., 2008).

The increase of mitochondrial biogenesis, hallmarked by a characteristic increase in cellular mtDNA level (Giordano et al., 2014), is a typical compensatory response to gross mitochondrial dysfunction (Lee et al., 2000) and has been reported in mitochondrial disorders characterized by a complex I defect (Giordano et al., 2014).

Here, we studied the mtDNA content in multiple tissues from PD patients and matched control subjects, comparing mtDNA copy number to cardinal measurements of PD etiology. Our observations indicate that mitochondrial biogenesis, meditated through mtDNA copy number, is important in the development of PD. In addition, our data suggest that mtDNA copy number may be a viable diagnostic predictor of PD.

## 2 Materials and methods

### 2.1 Patient cohort

We studied the role of mtDNA copy number in n = 363 peripheral blood samples, n = 151 substantia nigra pars compacta (SNpc) tissue samples, and n = 120 frontal cortex tissue samples from community-based PD cases fulfilling UK-PD Society brain bank criteria for the diagnosis of PD (Hughes et al., 1992), comparing the results to matched control samples with no clinical evidence of PD (n = 262 peripheral blood samples, n = 33 SNpc tissue samples, and n = 37 frontal cortex tissue samples). All were of Caucasian origin. All blood samples underwent cognitive assessment, including global cognitive function was assessed using the mini-mental state examination (MMSE) (Roth et al., 1986) and the Montreal Cognitive Assessment (Nasreddine et al., 2005). Cognitive impairment was determined using published criteria (using 1.5 standard deviation as a cutoff) (Yarnall et al., 2014). In addition, samples were genotyped for variants known to affective cognitive function: rs9468 (defining H1/H2, *MAPT*) and rs429358/rs7142 (defining *APOE1-4*).(Nombela et al., 2014) Genotyping was performed by KASP genotyping (LGC, Middlesex, UK).

### 2.2 MtDNA copy number determination

Total DNA was extracted from blood and tissue samples using standard methods. Quantification of mtDNA was performed in triplicate by multiplex Taqman qPCR amplification of the mitochondrial genes *MTND1* and *MTND4* and the nuclear encoded gene *B2M*, using serial dilutions of cloned vectors to ensure reaction linearity and for standard curve quantification, as previously detailed (Grady et al., 2014). MtDNA copy number is expressed as a relative abundance of both *MTND1* and *MTND4* to the nuclear counterpart, with a correlation of r^2^ = 0.9941 (MTND1:MTND4 derived copy number). This method was preferred to absolute (or standard curve) approaches as the low amount of DNA from microdissected tissues does not allow appropriate standard curve preparation (Giordano et al., 2014). Patient and control samples were randomly assigned to each run to limit run-specific stratification.

### 2.3 Validation of qPCR results

A random proportion (10%) of samples was replicated from source DNA, with a coefficient or repeat variance estimated as 0.3% (indicating that qPCR results are reproducible).

### 2.4 Statistical analysis

Data were analyzed using SPSS v22 with data-appropriate tests (detailed in text). Statistical significance was set at *p* ≤ 0.05.

### 2.5 Ethics

Local study approval was granted (NRES Committee North East-Newcastle & North Tyneside 1).

## 3 Results

**Fig. 1:**
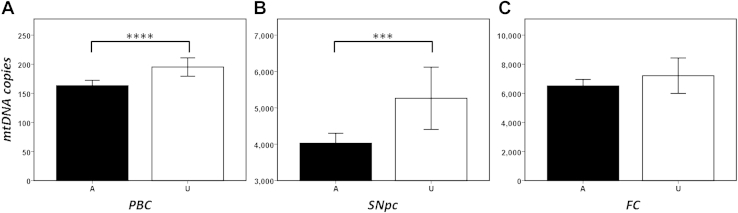
Analysis of mitochondrial DNA (mtDNA) content in 3 tissues, showing mean mtDNA copy number per cell (with 95% CI) for PD cases (A, shaded) and matched controls (C, unshaded) from (A) peripheral blood cells (PBC), (B) substantia nigra pars compacta (SNpc), and (C) frontal cortex (FC). Asterisks indicate statistical significance (****p = 10^−4^ and ***p = 10^−3^). Abbreviation: PD, Parkinson's disease.

We initially investigated the role of mtDNA copy number in peripheral white blood cells (PBCs), identifying a significant reduction in mtDNA copy number in PD cases when compared to controls (case mean = 163 ± 89, control mean = 195 ± 129, Mann-Whitney *p* = 2.86 × 10^−4^, Fig. 1A). This association was independent of age (linear regression PD vs. controls with age as a covariate, *p* = 3.0 × 10^−4^) and gender (males only, A = 227 vs. U = 129, Mann-Whitney *p* = 5.3 × 10^−4^ and females only, A = 133 vs. U = 132, Mann-Whitney *p* = 4.8 × 10^−2^).

Stratification to affected individuals only failed to find a significant association between PBC mtDNA copy number and phenotypic assessments, including dementia (Mann-Whitney *p* = 6.7 × 10^−1^), cognitive impairment (Mann-Whitney *p* = 3.9 × 10^−1^), MMSE score (Spearman correlation *p* = 6.85 × 10^−1^), Montreal Cognitive Assessment score (Spearman correlation *p* = 1.79 × 10^−1^), power of attention (Spearman correlation *p* = 8.27 × 10^−1^), continuity of attention (Spearman correlation *p* = 6.90 × 10^−1^), and cognitive reaction time (Spearman correlation *p* = 4.96 × 10^−1^; Table 1). Interestingly, we were able to identify a significant association between reduced PBC mtDNA copy number and smoking history (defined as current smoker or smoked <5 y from disease onset) in PD cases (Mann-Whitney *p* = 4.0 × 10^−3^, Table 1); however, we found no direct link between smoking history and PD (case/control *p* = 2.9 × 10^−1^).

Subsequent analysis of isolated brain tissue revealed a similar reduction of mtDNA copy number in PD cases, statistically significant in the vulnerable SNpc (Mann-Whitney U *p* = 4.0 × 10^−3^, Fig. 1B), but not in the asymptomatic FC (Mann-Whitney U *p* = 2.91 × 10^−1^, Fig. 1C). Substratification by gender revealed a significant association between SNpc mtDNA copy number in males (males only, A = 100 vs. U = 25, Mann-Whitney *p* = 5.0 × 10^−4^), but not in females (A = 49 vs. U = 8, Mann-Whitney *p* = 3.97 × 10^−1^); likely a result of low female sample numbers. We found no significant gender effect in FC mtDNA copy number. Correcting for age, as a covariate in linear regression, supported the association in SNpc (*p* = 4.5 × 10^−3^) and did not affect the result in FC (*p* = 8.4 × 10^−2^). In addition, we found no association between SNpc or FC tissue mtDNA copy number and dementia, MMSE, cognitive impairment, or smoking (Table 1). However, again, this is likely a direct result of a smaller sample number.

Despite reported links between inherited mtDNA variation and mtDNA copy number, mediated through respiratory chain coupling and reactive oxygen species,(Gomez-Duran et al., 2012, Liou et al., 2010) we found no link between mtDNA copy number and mtDNA haplogroup affiliation in PBC, SNpc, or FC tissue, when analyzed as separately or combined (cases + control; Table 1).

## 4 Discussion

In the largest study in PD to date, our observations indicate that mtDNA copy number is an important component in the pathoetiology of PD. Using different tissues, we have demonstrated that in high-turnover tissues such as PBCs and in vulnerable postmortem tissues such as the SNpc, mtDNA copy number is reduced and may be an important early biomarker for PD onset. Our results are in-keeping with similar reductions in mtDNA copy number reported in a number of other age-related neurological disorders where mitochondrial dysfunction was also a hallmark; For example, a PBC copy-number reduction in Huntington's disease (Petersen et al., 2014) and a reduction of mtDNA in pyramidal neurons in Alzheimer's disease hippocampi (Rice et al., 2014).

Interestingly, we found no link between cognitive phenotypes and mtDNA copy number, but our data indicate that smoking may be a factor in the reduction of mtDNA copy number in PD cases, supported by previous reports that cigarette smoke is linked to a reduction in PBC mtDNA (Xie et al., 2013). We also find no link between mtDNA an age of onset; however, this is likely a product of a limited age range of our samples (mean age—PBC = 69.1 StDev = 8.8, SNpc = 76.0 StDev = 8.5, and FC = 76.1 StDev = 10.0) and the fact that mitochondrial dysfunction in PD occurs during the early stages of neurodegeneration (Morais and De Strooper, 2010).

*What limitations can we identify?* We cannot rule out the effect of pharmacokinetics. A reduction in hippocampal and PBC mtDNA copy number was reported in chronic opiate abusers, mediated through autophagy (Feng et al., 2013). The complex and multifaceted nature of PD drug treatment makes these observations difficult to test and would require significantly larger sample numbers. Most of our observations are made in mtDNA isolated from peripheral blood, where differential blood composition (i.e., varying leukocyte count) could stochastically confound our results. However, as cell population of blood is highly regulated and we report mtDNA copy number corrected by cell number (using a housekeeping gene), we believe this is not an issue. In addition, we cannot completely control for is cell loss. Postmortem assessment of neuronal cell death will be anecdotal at best, and although studying isolated postmortem SNpc, we cannot completely rule out glial contamination in PD cases, where a loss of most neurons has occurred. However, glial contamination alone could not explain the gross mtDNA copy-number differences seen between cases and age-matched controls in the SNpc, moreover in the effect in PBCs.

Measuring mtDNA content in cells and tissues is not without problem. Extraction techniques affect template availability and can affect downstream processes, with varying mtDNA sequence affecting specificity. We mitigated these issues, using an established, internally correcting triplex probe-based approach (Grady et al., 2014); however, it is important that this effect is replicated in a similar cohort of PD patients and potentially expanded to a range of neurodegenerative diseases.

Finally, our observations are based on a single experimental series and, although our PBC and SNpc data are complimentary, we cannot wholly determine if this phenomenon is cause or effect or indeed limited to the brain regions under study. To address this further, we recommend longitudinal studies in PD patients, preferably investigating further vulnerable (i.e., cingulate) and unaffected brain regions.

## 5 Conclusion

Accepting our limitations, our study indicates that reduced mtDNA copy number in PBCs, supported by a subsequent reduction in the affected brain region, is a viable biomarker for the detection of PD.

## Disclosure statement

The authors declare that they have no competing financial interests.

## Figures and Tables

**Table 1:** Analysis of mtDNA copy number to key PD-related phenotypes, smoking history, and mtDNA haplogroup in PD cases

Samples	Tissue		
Blood	SNpc	FC
n = 363	n = 151	n = 120
mtDNA copy number versus			
Dementia*	6.87 × 10^-1^	4.70 × 10^-1^	3.48 × 10^-1^
Cognitive impairment*	3.91 × 10^-1^	5.32 × 10^-1^	6.54 × 10^-1^
MMSE**	6.85 × 10^-1^	6.15 × 10^-1^	1.65 × 10^-1^
MoCA**	1.79 × 10^-1^	—	—
Power of attention**	8.27 × 10^-1^	—	—
Continuity of attention**	6.90 × 10^-1^	—	—
Cognitive reaction time**	4.96 × 10^-1^	—	—
Smoking*	4.10 × 10^-3^	7.29 × 10^-1^	9.87 × 10^-1^
mtDNA haplogroup***	6.87 × 10^-1^	6.02 × 10^-1^	8.45 × 10^-1^

* Testing by Mann-Whitney *U* of mtDNA copy number versus binary variable, ** Testing by Spearman correlation of mtDNA copy number versus linear variable, and *** ANOVA testing of mtDNA copy number versus categorical variable.

Key: ANOVA, analysis of variance; FC, frontal cortex; MoCA, Montreal Cognitive Assessment; MMSE, mini-mental state examination; mtDNA, mitochondrial DNA; PD, Parkinson's disease; SNpc, substantia nigra pars compacta.

## References

[bib1] Davis G.C., Williams A.C., Markey S.P., Ebert M.H., Caine E.D., Reichert C.M., Kopin I.J. (1979). Chronic parkinsonism secondary to intravenous injection of meperidine analogues. Psychiatry Res..

[bib2] Feng Y.M., Jia Y.F., Su L.Y., Wang D., Lv L., Xu L., Yao Y.G. (2013). Decreased mitochondrial DNA copy number in the hippocampus and peripheral blood during opiate addiction is mediated by autophagy and can be salvaged by melatonin. Autophagy.

[bib3] Ghezzi D., Marelli C., Achilli A., Goldwurm S., Pezzoli G., Barone P., Pellecchia M.T., Stanzione P., Brusa L., Bentivoglio A.R., Bonuccelli U., Petrozzi L., Abbruzzese G., Marchese R., Cortelli P., Grimaldi D., Martinelli P., Ferrarese C., Garavaglia B., Sangiorgi S., Carelli V., Torroni A., Albanese A., Zeviani M. (2005). Mitochondrial DNA haplogroup K is associated with a lower risk of Parkinson's disease in Italians. Eur. J. Hum. Genet..

[bib4] Giordano C., Iommarini L., Giordano L., Maresca A., Pisano A., Valentino M.L., Caporali L., Liguori R., Deceglie S., Roberti M., Fanelli F., Fracasso F., Ross-Cisneros F.N., D'Adamo P., Hudson G., Pyle A., Yu-Wai-Man P., Chinnery P.F., Zeviani M., Salomao S.R., Berezovsky A., Belfort R., Ventura D.F., Moraes M., Moraes Filho M., Barboni P., Sadun F., De Negri A., Sadun A.A., Tancredi A., Mancini M., d'Amati G., Loguercio Polosa P., Cantatore P., Carelli V. (2014). Efficient mitochondrial biogenesis drives incomplete penetrance in Leber's hereditary optic neuropathy. Brain.

[bib5] Gomez-Duran A., Pacheu-Grau D., Martinez-Romero I., Lopez-Gallardo E., Lopez-Perez M.J., Montoya J., Ruiz-Pesini E. (2012). Oxidative phosphorylation differences between mitochondrial DNA haplogroups modify the risk of Leber's hereditary optic neuropathy. Bba-mol Basis Dis..

[bib6] Grady J.P., Murphy J.L., Blakely E.L., Haller R.G., Taylor R.W., Turnbull D.M., Tuppen H.A.L. (2014). Accurate measurement of mitochondrial DNA deletion level and copy number differences in human skeletal muscle. PLoS One.

[bib7] Hatano Y., Li Y., Sato K., Asakawa S., Yamamura Y., Tomiyama H., Yoshino H., Asahina M., Kobayashi S., Hassin-Baer S., Lu C.S., Ng A.R., Rosales R.L., Shimizu N., Toda T., Mizuno Y., Hattori N. (2004). Novel PINK1 mutations in early-onset parkinsonism. Ann. Neurol..

[bib8] Hudson G., Gomez-Duran A., Wilson I.J., Chinnery P.F. (2014). Recent mitochondrial DNA mutations increase the risk of developing common late-onset human diseases. PLoS Genet..

[bib9] Hudson G., Nalls M., Evans J.R., Breen D.P., Winder-Rhodes S., Morrison K.E., Morris H.R., Williams-Gray C.H., Barker R.A., Singleton A.B., Hardy J., Wood N.E., Burn D.J., Chinnery P.F. (2013). Two-stage association study and meta-analysis of mitochondrial DNA variants in Parkinson disease. Neurology.

[bib10] Hughes A.J., Daniel S.E., Kilford L., Lees A.J. (1992). Accuracy of clinical diagnosis of idiopathic Parkinson's disease: a clinico-pathological study of 100 cases. J. Neurol. Neurosurg. Psychiatry.

[bib11] Latsoudis H., Spanaki C., Chlouverakis G., Plaitakis A. (2008). Mitochondrial DNA polymorphisms and haplogroups in Parkinson's disease and control individuals with a similar genetic background. J. Hum. Genet..

[bib12] Lee H.C., Yin P.H., Lu C.Y., Chi C.W., Wei Y.H. (2000). Increase of mitochondria and mitochondrial DNA in response to oxidative stress in human cells. Biochem. J..

[bib13] Liou C.W., Lin T.K., Chen J.B., Tiao M.M., Weng S.W., Chen S.D., Chuang Y.C., Chuang J.H., Wang P.W. (2010). Association between a common mitochondrial DNA D-loop polycytosine variant and alteration of mitochondrial copy number in human peripheral blood cells. J. Med. Genet..

[bib14] Mills R.D., Sim C.H., Mok S.S., Mulhern T.D., Culvenor J.G., Cheng H.C. (2008). Biochemical aspects of the neuroprotective mechanism of PTEN-induced kinase-1 (PINK1). J. Neurochem..

[bib15] Morais V.A., De Strooper B. (2010). Mitochondria dysfunction and neurodegenerative disorders: cause or consequence. J. Alzheimers Dis..

[bib16] Mullin S., Schapira A. (2013). Alpha-Synuclein and mitochondrial dysfunction in Parkinson's disease. Mol. Neurobiol..

[bib17] Nasreddine Z.S., Phillips N.A., Bedirian V., Charbonneau S., Whitehead V., Collin I., Cummings J.L., Chertkow H. (2005). The Montreal Cognitive Assessment, MoCA: a brief screening tool for mild cognitive impairment. J. Am. Geriatr. Soc..

[bib18] Nombela C., Rowe J.B., Winder-Rhodes S.E., Hampshire A., Owen A.M., Breen D.P., Duncan G.W., Khoo T.K., Yarnall A.J., Firbank M.J., Chinnery P.F., Robbins T.W., O'Brien J.T., Brooks D.J., Burn D.J., Barker R.A., ICICLE-PD Study Group (2014). Genetic impact on cognition and brain function in newly diagnosed Parkinson's disease: ICICLE-PD study. Brain.

[bib19] Petersen M.H., Budtz-Jorgensen E., Sorensen S.A., Nielsen J.E., Hjermind L.E., Vinther-Jensen T., Nielsen S.M.B., Norremolle A. (2014). Reduction in mitochondrial DNA copy number in peripheral leukocytes after onset of Huntington's disease. Mitochondrion.

[bib20] Reeve A., Meagher M., Lax N., Simcox E., Hepplewhite P., Jaros E., Turnbull D. (2013). The impact of pathogenic mitochondrial DNA mutations on substantia nigra neurons. J. Neurosci..

[bib21] Rice A.C., Keeney P.M., Algarzae N.K., Ladd A.C., Thomas R.R., Bennett J.P. (2014). Mitochondrial DNA copy numbers in pyramidal neurons are decreased and mitochondrial biogenesis transcriptome signaling is disrupted in Alzheimer's disease hippocampi. J. Alzheimers Dis..

[bib22] Roth M., Tym E., Mountjoy C.Q., Huppert F.A., Hendrie H., Verma S., Goddard R. (1986). CAMDEX. A standardised instrument for the diagnosis of mental disorder in the elderly with special reference to the early detection of dementia. Br. J. Psychiatry.

[bib23] Xie H., Lev D., Gong Y.L., Wang S., Pollock R.E., Wu X.F., Gu J. (2013). Reduced mitochondrial DNA copy number in peripheral blood leukocytes increases the risk of soft tissue sarcoma. Carcinogenesis.

[bib24] Yarnall A.J., Breen D.P., Duncan G.W., Khoo T.K., Coleman S.Y., Firbank M.J., Nombela C., Winder-Rhodes S., Evans J.R., Rowe J.B., Mollenhauer B., Kruse N., Hudson G., Chinnery P.F., O'Brien J.T., Robbins T.W., Wesnes K., Brooks D.J., Barker R.A., Burn D.J., Group I.-P.S. (2014). Characterizing mild cognitive impairment in incident Parkinson disease: the ICICLE-PD study. Neurology.

